# Pharmacokinetic, Metabolism, and Metabolomic Strategies Provide Deep Insight Into the Underlying Mechanism of *Ginkgo biloba* Flavonoids in the Treatment of Cardiovascular Disease

**DOI:** 10.3389/fnut.2022.857370

**Published:** 2022-03-23

**Authors:** Yi Tao, Fei Zhu, Meiling Pan, Qing Liu, Ping Wang

**Affiliations:** College of Pharmaceutical Science, Zhejiang University of Technology, Hangzhou, China

**Keywords:** *Ginkgo biloba*, flavonoids, pharmacokinetics, metabolism, metabolomics, cardiovascular disease, gut microbiota

## Abstract

*Ginkgo biloba*, known as the “living fossil,” has a long history of being used as botanical drug for treating cardiovascular diseases and the content of flavonoids as high as 24%. More than 110 different kinds of flavonoids and their derivatives have been separated from *G. biloba*, including flavones, flavonols, biflavonoids, catechins, and their glycosides, etc., all of which display the ability to dilate blood vessels, regulate blood lipids, and antagonize platelet activating factor, and protect against ischemic damage. At present, many types of preparations based on *G. biloba* extract or the bioactive flavonoids of it have been developed, which are mostly used for the treatment of cardiovascular diseases. We herein review recent progress in understanding the metabolic regulatory processes and gene regulation of cellular metabolism in cardiovascular diseases of *G. biloba* flavonoids. First, we present the cardioprotective flavonoids of *G. biloba* and their possible pharmacological mechanism. Then, it is the pharmacokinetic and liver and gut microbial metabolism pathways that enable the flavonoids to reach the target organ to exert effect that is analyzed. In the end, we review the possible endogenous pathways toward restoring lipid metabolism and energy metabolism as well as detail novel metabolomic methods for probing the cardioprotective effect of flavonoids of *G. biloba*.

## Introduction

Cardiovascular disease is one type of diseases with the highest morbidity and mortality worldwide, mainly including angina pectoris, hypertension, hyperlipidemia, atherosclerosis, and stroke (1). Cardiovascular disease brings heavy economic burden on patients and makes it urgent to prevent and treat the disease (2). Botanical drugs, such as *Ginkgo biloba*, are effective in prevention of cardiovascular disease.

As an herb belongs to the Ginkgoaceae, *G. biloba*, which has a long history of medicinal use (3), has been recorded to own the effects of promoting blood circulation, removing blood stasis, dredging collaterals, relieving pain, and lowering lipids. Since the 1960s, German scientists discovered for the first time that *G. biloba* contained medicinal ingredients for the treatment of cardiovascular diseases (4). After that, *G. biloba* extract has attracted more and more attention all over the world. One of the main key components in *G. biloba* extract is flavonoids, the content of which is as high as 24%. In several epidemiological studies, dietary flavonol and flavonoid intake was inversely associated with the risk of cardiovascular disease (5). Current pharmacological investigations have revealed that the flavonoids extracted from *G. biloba* has prominent cardioprotective activities such as dilating blood vessels (6, 7), regulating blood lipids (8), lowering blood sugar (9), inhibiting cardiomyocyte apoptosis (10, 11) and preventing myocardial ischemic injury (12, 13) and vascular rupture (14).

**FIGURE 1:**
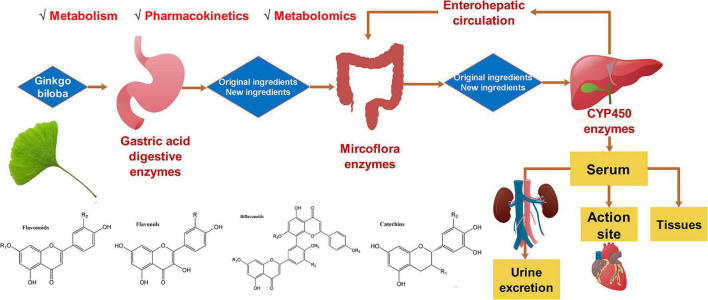
The metabolism, pharmacokinetics, and metabolomics of *Ginkgo biloba* flavonoids.

A plethora of *G. biloba* flavonoids have been separated, purified, and identified. To date, more than 110 flavonoids and their derivatives are isolated from ginkgo leaves (15). According to the chemical structures, flavonoids are mainly divided into four categories: flavones, flavonols, biflavonoids, catechins, and their glycosides (16). As is shown in Figure 1, this review focused on the progress of pharmacokinetics, metabolism, and metabolomics of *G. biloba* flavonoids in recent years, which will shed light on the cardioprotective mechanism of *G. biloba* flavonoids and lay a foundation for clinical use of the *G. biloba* flavonoids.

## Cardioprotective Flavonoids of *Ginkgo Biloba*

The flavones in *G. biloba* mainly include apigenin, luteolin, and chrysanthin, the cardioprotective mechanism of which were summarized in Supplementary Table 1. Apigenin can protect vascular endothelial (17, 18), reduce myocardial damage (19, 20), serve as anticoagulant (21), and prevent aortic aneurysm (22). Not only could apigenin improve the functional recovery of ischemic heart through PI3K/Akt pathway and reduce myocardial infarction size, but it also could downregulate the activities of creatine kinase isoenzyme and lactate dehydrogenase in coronary blood flow, and minimize the number of apoptotic cardiomyocytes. In addition, apigenin protected myoblast H9c2 from ischemia/hypoxia-induced myocardial injury and inhibited the expression of pro-inflammatory factors (23). Apigenin significantly prevented platelet aggregation at a concentration of 2500 mM *in vitro*, however, since this concentration was difficult to achieve *in vivo*, consuming large amounts of apigenin-rich foods would not affect platelet aggregation nor other hemostatic variables in healthy volunteers (24). Several studies have shown that luteolin has beneficial effects on cardiovascular diseases, such as reducing atherosclerosis (25, 26), protecting vascular endothelial cells (27, 28), improving hypertension complications (29), and dilating coronary arteries (30). For instance, due to that luteolin interacted with activator factor 3 (STAT3) mainly through hydrogen bonding interactions, luteolin reduced oxLDL-induced inflammation by inhibiting STAT3 signaling and transcription (26). Moreover, by inducing NO production and reducing oxidative stress (31), luteolin protected venous endothelial cells. By promoting signaling of the endogenous antioxidant enzyme peroxidase II, increasing the expression of the anti-apoptotic protein Bcl-2 and decreasing the expression of the pro-apoptotic protein Bax (32, 33), it ameliorated myocardial ischemia-reperfusion injury.

Flavonols of *G. biloba* contain quercetin, kaempferol, and isorhamnetin. The monoglycosides of flavonols are mainly glucoside and isorhamnoside (34). The flavonols of *G. biloba* have cardioprotective, antioxidant, antibacterial, and neuroprotective effects (35). The effect of flavonol of *G. biloba* on improving blood circulation may be partly attributed to its promotion of thrombomodulin expression and tissue-type plasminogen activator (t-PA) secretion in endothelial cells, and quercetin is heavily involved in promotion effect on t-PA secretion (36). Meanwhile, one study (37) showed that flavonol of *G. biloba* exhibited a concentration-dependent vasodilation effect. Rat aortic annulus showed strong contraction after initial application of 5 μM norepinephrine (NE). Subsequent application of flavonol of *G. biloba* (0.03–3 g L^–1^) effectively relaxed NE-induced contractions in a concentration-dependent manner. Quercetin produced a significant vasodilation effect at a concentration of 0.1 μM, whereas 100 μM of quercetin can cause a very strong vasodilation effect, and the vasodilation rate reached 49.9 ± 4.8%. Of note, kaempferol can significantly enhance vascular endothelial cell proliferation, migration, and angiogenesis by binding to vascular endothelial growth factor (VEGF) (38).

Biflavonoids are a class of compounds formed by the polymerization of two flavonoid cores through C-C bonds, and are usually characteristic chemical components of gymnosperms. So far, thirteen biflavonoids have been isolated and identified from *G. biloba* leaves, including ginkgetin, 7′-O-β-D-glucosyl-ginkgetin, isoginkgetin, 7′-O-β-D-glucosyl-isoginkgetin, 2,3-dihydroisoginkgetin, 2,3-dihydrosciadopitysin, amentoflavone, bilobetin, sesquojaflavone, podocarpusflavone A, 7-methoxy- amentoflavone, 5′-methoxybilobetin, and sciadopitysin. These biflavonoids have a variety of pharmacological effects, such as cardioprotective, anti-inflammatory, antioxidant, and neuroprotection. One work (39) established a DPPH/ABTS free radical scavenging model and the nitric oxide measurement method to find that amentoflavone own oxygen free radicals scavenging effect in a dose-dependent manner. Moreover, another work (40) discovered that four biflavonoids (i.e., ginkgetin, isoginkgetin, bilobetin, and amentoflavone) can occupy the active hole of human thrombin, bind to the exosite I to inhibit the activity of thrombin and prevent the formation of thrombus. cAMP-phosphodiesterases (cAMP-PDEs) have a regulatory effect on cardiac excitation-contraction coupling. Interventions targeting cAMP-PDEs have important implications for the treatment of heart failure (41). It was reported that the inhibitory degree of bioflavonoids of *G. biloba* on this enzyme from high to low was ranked as below: amentoflavone > bilobetin > sesquojafla-vone > ginkgetin = isoginkgetin. Of note, sciadopitysin is almost inactive. It has been demonstrated that the inhibitory activity of biflavonoids is directly related to the number of free hydroxyl groups. Biflavonoids of *G. biloba* showed complete inhibition at a much lower concentration than the well-known cAMP-phosphodiesterase inhibitors papaverine, aminophylline, and kaempferol (42, 43).

Catechins of *G. biloba* are divided into four types: catechins, epicatechins, gallic acid catechins, and epigallic acid catechins. In addition, there are dimers of 4, 8′′-catechin gallate catechins and 4, 8′′-gallate catechin gallate catechins. Wu et al. (44) reported that epigallocatechin inhibited endoplasmic reticulum stress-related thioredoxin-interacting protein (TXNIP) and NOD-like receptor protein 3 (NLRP3) inflammasome activation, thereby protecting endothelial cells from inflammatory and apoptotic damage.

## Pharmacokinetics of Flavonoids of *Ginkgo Biloba*

Unlike terpenes with high bioavailability, flavonoids have extremely low bioavailability due to the extensive first pass effect and glucuronidation (45). Flavonoids of *G. biloba* are mainly absorbed in the form of aglycon, and mainly exist in the form of glucuronate or sulfate in plasma and urine (46–49). One work (50) labeled *G. biloba* preparation with radioactive element ^14^C and administered them to the rats. It is showed in the result that with a first-order elimination rate and a half-life of about 4.5 h, the pharmacokinetic of *G. biloba* preparation fitted to a two-compartment model. *G. biloba* was absorbed through the gastrointestinal tract after oral administration, and the absorption rate was more than 60%. The amount of ^14^C-CO_2_ exhaled accounted for 38% of the dose after 72 h of oral administration. 22% of the dose is excreted in urine, while 29% of the dose is excreted in feces. The glands, neuron tissues, and eyes show high affinity for the labeled *G. biloba* preparation.

Another group (51) developed and validated a novel liquid chromatography-tandem mass spectrometry method using dynamic multiple reaction monitoring (DMRM) for the simultaneous determination of three flavonoids (i.e., quercetin, kaempferol, isorhamnetin) of *G. biloba* extract in rat plasma. Compared to traditional multiple reaction monitoring (MRM), DMRM reduces the number of concurrent MRM transitions and increases dwell time significantly, and provides greater sensitivity.

Ten adult volunteers with an average age of 28 years were each given a single oral dose of six tablets of *G. biloba* extract at a time (52). Reversed-phase high performance liquid chromatography was applied to determine the levels of quercetin and kaempferol in human urine at different periods. The elimination rate constant K_e_ and absorption rate constant K_a_ of quercetin were slightly larger than that of kaempferol. In contrast, the absorption half-life (t_1/2a_), elimination half-life (t_1/2_) and t_max_ of quercetin were all smaller than that of kaempferol. The mean values of K_a_ were 0.61 and 0.55 h^–1^ for quercetin and kaempferol, respectively. As a result, quercetin and kaempferol are mainly excreted in human urine in the form of glucuronide.

Extensive first-pass metabolism is believed to be the main cause for the low bioavailability of flavonoids, apart from which, P-glycoprotein (P-gp)-mediated efflux is another reason for the low bioavailability of *G. biloba* flavonoids. As an ATP-driven efflux pump, P-gp can transport a wide variety of compounds of various structures from the interior of the cell into the extracellular space. P-gp exists in both tumor cells and normal tissues, and it plays an important role in the process of drug absorption in the human body. Quercetin, kaempferol and isorhamnetin are substrates of P-gp. P-glycoprotein-type efflux pumps may limit the bioavailability of ginkgo flavonols (53). In the presence of breast cancer resistance protein inhibitors, the intracellular concentration of kaempferol was found to increase significantly (54). Moreover, the sulfation of intestinal metabolites and the activity of efflux transporters can form a connecting barrier to jointly prevent flavonoid aglycones from entering the portal vein and circulation and to reduce their absorption.

The components of *G. biloba* extracts are complex. Compared with that of a single compound, the synergistic effects of coexisting components can inevitably induce interactions between the components and change their pharmacokinetic behaviors. One group (55) investigated the pharmacokinetic parameters of *G. biloba* extract and ginkgo flavonoids after oral administration. Compared with those of the ginkgo flavonoids treated group, the C_max_, AUC_0 → t_ values and absorption rate of all analytes (except for the C_max_ of naringenin) were significantly improved in the *G. biloba* extract treated group. As is mentioned earlier, intestinal efflux mediated by transporters may contribute to the low bioavailability of ginkgo flavonoids. It is worth noting that in the presence of kaempferol, the transport rate of quercetin efflux by MDCK/Bcrp1 cells decreased by 11.6 times (from 97.5 to 8.37), which implied that kaempferol could inhibit the efflux of quercetin. Further investigation revealed that kaempferol, quercetin and isorhamnetin had strong mutual inhibition on efflux mediated by P-glycoprotein transporters. The K_i_ values of kaempferol to quercetin and isorhamnetin are determined to be 4.64 ± 3.45 and 18.42 ± 3.87 μM, respectively, while the K_i_ values of quercetin to kaempferin and isorhamnetin are calculated as 11.10 ± 0.30 and 2.26 ± 0.99 μM, respectively. Moreover, the K_i_ values of isorhamnetin to kaempferol and quercetin are 24.99 ± 2.87 and 5.27 ± 2.40 μM. The interactions between the components may facilitate the absorption of flavonoids of *G. biloba*.

Collectively, most of the ginkgo flavonoids are hydrolyzed under the action of cytosolic β-glucosidase (CBG) in the small intestine, and then absorbed in the form of aglycones after hydrolysis. This procedure is a key step in the absorption and metabolism of flavonoid glycosides (47). Then, flavonoid aglycones usually pass through the intestinal wall and enter the intestinal epithelial cells, and finally reach the liver through the portal vein or the portal chyle duct. In the liver, flavonoid aglycones first undergo phase I metabolism such as hydroxylation of liver cytochrome P450. Phase I metabolism contributed little to metabolism of flavonoid aglycones. On the contrary, phase II metabolism played a pivotal role in metabolism of flavonoid aglycones. At present, there are mainly three types of phase II metabolic enzymes, which are involved in the metabolism of flavonoid aglycones, including uridine 5′-diphospho-glucuronosyl transferase (UGT), sulfotransferase (SULT), and catechol-O-methyltransferase (COMT) (56). Under the action of UGT, SULT, and COMT, flavonoid aglycones can undergo glucuronidation, sulfation, and methylation reactions and generate corresponding glucuronides, sulfates, and methylated metabolites. The typical bimodal phenomenon of ginkgo flavonoids is caused by enterohepatic circulation. Some flavonoid glycosides were rapidly absorbed in the upper part of the digestive tracts and can be excreted through the biliary tracts, which entered the gut again and reabsorbed. This phenomenon will help to increase serum levels of flavonoids and prolong their half-life time.

## Metabolism of Flavonoids of *Ginkgo Biloba* by Liver

Flavonoid glycosides of *G. biloba* are mainly metabolized in two parts of the body: one is the liver, where a series of reactions occur under the action of liver CYP450 to produce metabolites with higher water solubility; the second is the intestinal tract, where flavonoid glycosides are hydrolyzed into aglycon under fermentation of intestinal flora. Cytochrome P450 (CYP450) enzyme system is the major enzyme system of liver for drug metabolism, which has a wide range of biological significance. CYP450 enzyme system mainly includes CYP1A2, CYP2A6, CYP2B6, CYP2C, CYP2D6, CYP2E1, and CYP3A. In the liver, flavonoids of *G. biloba* are mainly subjected to phase II metabolism, and their main metabolic pathway is glucuronic acid binding reaction. The liver metabolism pathway of quercetin is presented in Supplementary Figure 1. The major enzyme that mediates their phase II metabolism is UGT1A9 (57, 58). *G. biloba* extract can inhibit the activity of UGT1A9 (59, 60). Meanwhile, flavonoids of *G. biloba* also exert effect on the expression of phase II metabolizing enzymes (61–69) (see Table 1). A serum concentration of about 100 nM for luteolin can be reached by dietary habits, but less than 1 nM luteolin was capable of inducing the expression of phase II drug metabolizing enzymes through the ERK1/2 signaling pathway, such as glutathione S-transferase (GST), heme oxygenase-1 (HO-1), NAD(P)H: quinone oxidoreductase 1 (NQO1), and aldo-keto reductase family 1 member B10 (AKR1B10), increasing the stability of Nuclear factor-erythroid-2-related factor 2 (Nrf2) and inducing conformational changes in Kelch-like EXH-associated protein 1 (Keap1) (70).

**TABLE 1:** Impact of flavonoids of *Ginkgo biloba* on metabolism enzymes.

Sample	Specific metabolism enzyme	Model	Impact effect	References
GBE	CYP2B1 CYP3A23	hepatocytes	activation inhibition	(61)
GBE	CYP3A4	liver microsomes	activate expression inhibit activity	(62)
GBE	CYP2B6	liver microsomes	inhibition	(63)
GBE	CYP1A1	Caco-2 cells	inhibition	(64)
GBE	CYP3A4 CYP2D6	liver microsomes	inhibition	(65)
GBE	CYP3A	intestinal mucosa	inhibition	(66)
Quercetin	CYP1A2 CYP2A6	liver microsomes	inhibition activation	(67)
Quercetin	CYP 3A	liver microsomes	inhibition	(68)
Luteolin	CYP3A4 CYP3A5	liver microsomes	inhibition inhibition	(69)
Luteolin quercetin	β-glucuronidase	gut microbiota	inhibition	(75)
Amentoflavone	β-glucuronidase	gut microbiota	inhibition	(76)

On the one hand, CYP450 enzymes and intestinal flora can metabolize flavonoids of *G. biloba* (71). On the other hand, flavonoids of *G. biloba* also affect the activity of CYP450 enzymes and the abundance of intestinal flora. The current research pertaining to *G. biloba* flavonoids and CYP450 enzymes is mainly focused on CYP3A4, which is one of the key enzymes of drug metabolism and plays a pivotal role in the metabolism of more than 50% of the drugs on the market. It’s intriguing that many studies have shown that ginkgolides have the effect of activating CYP3A4 enzyme system, while *G. biloba* extract and flavonoids have inhibitory effects against CYP3A4 enzyme (62, 65, 66). However, one study has also shown that kaempferol has a weak effect on CYP3A4 enzyme (72). This phenomenon can be explained by the fact that kaempferol can activate the expression of CYP3A enzyme but inhibit its activity. Taken together, quercetin and kaempferol can reduce the enzyme activities of CYP1A1, CYP1A2, and CYP3A4 (64).

## Gut Microbiota and Flavnoids of *Ginkgo Biloba*

The gut microbiota is a collection of microorganisms living in the gastrointestinal tract that provides important signaling metabolites for the host (73, 74). After orally administered, flavonoids of *G. biloba* pass through the gastrointestinal tract and are transformed by the gut microbiota (75, 76). The transformation of *G. biloba* flavonoids by the gut microbiota is presented in Supplementary Figure 2. Lin et al. (77) investigated the effect of gut microbiota on the biotransformation of quercetin, kaempferol, luteolin, apigenin, and naringenin. To differentiate *in vitro* fecal fermentation of flavonoids from enzymatic or chemical degradation, flavonoids were incubated with fecal microbial suspensions or heat-killed intestinal microbial suspensions. Compared with the heat-inactivated group, all flavonoids in the fecal microbial suspension group were metabolized within 48 h of fermentation, suggesting that the loss of parent compounds in the activated suspension was ascribed to the enzymatic activity of the gut microbiota. Meanwhile, purified flavonoids were administered to control mice and antibiotic-treated mice by gavage, and the metabolism of these flavonoids was elucidated. The gut microbiota broke down the heterocycles of flavonoids and produced a series of phenolic metabolites. *p*-Hydroxyphenylacetic acid, protocatechuic acid, *p*-hydroxybenzoic acid, vanillic acid, hydrocaffeic acid, coumaric acid, and 3-(4-hydroxyphenyl)propionic acid were detected in serum samples of control group following oral consumption of these flavonoids. The transformation route was displayed in Supplementary Figure 2. Deglycosylation of flavonoids by β-glucosidase and endo-β-glucosidase occurs as the first step of metabolism, and then gut flora further breaks down and metabolizes aglycones to phenolic compounds. Phenylacetic acid and its hydroxylated forms are the main fermentation products. The released glycosyl moieties can be used as fermentation co-substrates, thereby accelerating the fermentation process of flavonoids. Compared with control group, a significantly lower concentrations of phenolic metabolites were observed in the antibiotic-treated group. A significantly higher concentration of flavonoids was excreted in feces and urine. These results suggested that the gut microbiota plays an important role in the metabolism and degradation of flavonoids of *G. biloba*.

The metabolism of gut microbiota may also affect the bioavailability of the flavonoids (78). Hanske et al. (79) investigated the effect of human gut microbiota on bioavailability of apigenin-7-glucoside (A7G) *in vitro* and in germ-free and human gut microbiota-associated (HMA) rats. Apigenin-7-O-glucoside was completely converted within 5 h after incubation with a fecal suspension containing the human gut microbiota. Apigenin and naringenin were transiently formed as intermediate metabolites, and the final degradation product was 3-(4-hydroxyphenyl)propionic acid (4-HPPA) and 3-(3-hydroxyphenyl)propionic acid (3-HPPA). In contrast, the concentration of A7G remained stable in germ-free group. These *in vitro* experiments demonstrated the ability of the human gut microbiota on transformation of A7G. After administration to germ-free rats, apigenin, luteolin and their conjugates were detected in urine and feces. While in HMA rats, 3-(4-hydroxyphenyl)propionic acid was observed as the major metabolite in urine. Specific gut microbiota was tested for their ability to deglycosylate A7G. Not all commensal gut microbiota were found to deglycosylate apigenin. Notably, cytoplasmic extracts of *Eubacterium* and *Bacteroides* were able to convert A7G to apigenin. Overall, this study suggested a crucial role of gut microbiota in the metabolism of apigenin.

On the other hand, flavonoids of *G. biloba* can modulate the structure and function of the intestinal flora. For instance, one work (80) cultivated hybrid groupers on a diet with *G. biloba* extract. The work found that dietary supplementation of 0.50–2.00 g/kg *G. biloba* extract improved gut morphology and increased expression of zonula occludens 1, zonula occludens 2, zonula occludens 3, occludin, and claudin 3a in hybrid grouper. Moreover, another work (81) found that apigenin can significantly improve intestinal dysbiosis induced by high-fat diet, increase the abundance of *Akkermansia*, *Incertae_Sedis* and reduce the abundance of *Faecalibaculum* and *Dubosiella* to restore intestinal barrier damage. In addition, *G. biloba* extract was evaluated for its potential as a feed additive for ruminant animals (82). The levels of total bacteria, *Ruminococcus flavefaciens*, *Ruminococcus albus*, and *Fibrobacter succinogenes* were decreased by administration of *G. biloba* extract, whereas the levels of *Selenomonas ruminantium*, *Anaerovibrio lipolytica*, *Ruminobacter amylophilus*, *Succinivibrio dextrinosolvens*, and *Megasphaera elsdenii* were increased by ginkgo extract supplementation, which led to the higher propionate production. Amentoflavone derived from *G. biloba* displayed relatively strong inhibition on three gut bacterial β-glucuronidases including CpGUS, SpasGUS, and EcGUS, which play an important role in deconjugation of various O-glucuronides (76). Intriguingly, treatment with *G. biloba* extract did not affect intestinal expression of human breast cancer resistance protein, but treatment with the lysates of *G. biloba* extract-treated mouse feces significantly suppressed expression of human breast cancer resistance protein (83). These results suggested that *G. biloba* extract changed the function of intestinal flora indeed.

## Impacting Factors on Bioavailability of Flavonoids of *Ginkgo Biloba*

The flavonoids of *G. biloba* mainly exist in the form of monoglycosides, diglycosides and triglycosides. One group (84) studied the effect of glycosylation on the absorption and metabolism of quercetin in rats. Four groups of rats were orally administered 20 mg of quercetin or equivalent quercetin 3-glucoside, or quercetin 3-rhamnoside or rutin. After 4 h, a HPLC-UV method was applied to determine the concentration of the flavonoids and its metabolites *via* glucuronidase or sulfatase in rat plasma. As a result, the quercetin metabolites in the plasma of rats in all groups were the same, but their total concentrations were quite different. The plasma level of the quercetin in quercetin treated group was 11.7 ± 1.8 μM, but when quercetin was administered as quercetin-3-*O*-glucoside, the level of quercetin was three times higher (33.2 ± 3.5 μM). In contrast, the plasma concentration of quercetin in the rutin treated group was very low (approximately 3 μM) and was even undetectable in the quercetin-3-*O*-rhamnoside treated group. These findings indicated that 3-*O*-glucosylation increases the absorption of quercetin in the small intestine, whereas the glycosides containing the rhamnose moiety significantly inhibits the absorption of rutin. Therefore, the nature of glycosylation significantly affects the absorption efficiency of quercetin in rats. In addition, one study (85) discovered three new compounds in *G. biloba* and compared their antioxidant activity with aglycone. It was worth noting that the antioxidant activity of aglycones was about three times higher than that of their respective glycosides, and about three times higher than that of positive control, i.e., ascorbic acid.

The oral bioavailability of a drug is closely related to its oral absorption route in the gastrointestinal tract. Part of the flavonoid glycosides are quickly absorbed in the upper digestive tract, and some of them are absorbed after being metabolized by the intestinal flora in the lower digestive tract. One group (86) investigated how the sugar group affected absorption of quercetin. When these compounds (i.e., quercetin glucoside and quercetin rutinoside) were fed to nine volunteers, the peak concentration of quercetin (C_max_) in plasma of quercetin glucoside-fed group was 20 times higher than that of quercetin rutinoside-fed group. Moreover, T_max_ of the former group was more than ten times shorter that the latter group. The bioavailability of the quercetin rutinoside was only 20% of that of the quercetin glucoside. This phenomenon suggests that quercetin glucoside is actively absorbed from the small intestine, whereas quercetin rutinoside is absorbed from the colon after deglycosylation.

Different administration methods have great influence on the bioavailability of flavonoids of *G. biloba*. One study (87) established an UPLC-Q-TOF-MS method to compare the metabolic profiles of amentoflavone (AMF), which were given by gavage (500 mg/kg) and intravenous injection (10 mg/kg) in rats. The oral bioavailability of AMF was only 0.06 ± 0.04%, and the area under the curve of the glucuronidated AMF metabolites (410.938 ± 62.219 ng/mL h) was significantly higher than that of AMF (194.509 ± 16.915 ng/mL h). Due to the poor solubility of flavonoids and poor absorption effect, the intravenous administration method can avoid the first pass effect, which greatly improves the bioavailability of flavonoids of *G. biloba*.

The state of the body also affects the biotransformation and metabolic absorption of flavonoids of *G. biloba*. One group (88) combined an offline hydrophilic interaction × reversed-phase two-dimensional liquid chromatography (HILIC × RP 2D-LC) system with diode array detection (DAD) and quadrupole time-of-flight mass spectrometry (Q/TOF-MS) for identifying and quantifying the biotransformation of flavonoids of *G. biloba* in normal and diabetic rat liver microsomes (RLM). Compared with normal RLMs, the metabolic rates of four target compounds, i.e., quercetin, genistein, kaempferol, and isorhamnetin, were significantly increased in diabetic RLMs. Enzyme kinetics investigation showed that the Michaelis-Menten constant (K_m_) value of genistein in diabetic RLMs was significantly increased as compared with normal RLMs, whereas its maximum velocity (V_max_) and intrinsic clearance (CL_int_) values were significantly decreased. In contrast, the CL_int_ values of kaempferol and isorhamnetin were significantly increased, and their K_m_ values were significantly decreased.

New drug delivery systems for flavonoids of *G. biloba* are also under development. One group (89) applied ultra-performance liquid chromatography-tandem mass spectrometry to compare the absorption and metabolism of *G. biloba* extract and its liposome preparations. Compared with the control group, the AUC_0 → t_ and C_max_ values of quercetin, kaempferol and isorhamnetin in the liposome treated group were significant increased. Liposome encapsulation improve the bioavailability of *G. biloba* extract. Moreover, another group (90) prepared phospholipid complex (GBP) and solid dispersion (GBS) of *G. biloba* extract, and compared their pharmacokinetic parameters and oral bioavailability. It was demonstrated that the bioavailability of quercetin, kaempferol, and isorhamnetin in the GBP and GBS groups increased significantly in comparison with the *G. biloba* extract group, and the bioavailability of GBP was higher than that of GBS.

## Metablomics on Flavnoids of *Ginkgo Biloba*

Dietary flavonoid intake is associated with a reduced risk of cardiovascular disease, possibly by affecting metabolic health. Different flavonoids have different effects on the energy and lipids metabolism. Apigenin, quercetin and epicatechin all significantly reduced high-fat diet-induced weight gain, of which quercetin was the most effective in reducing liver lipid accumulation by up to 70% (91, 92). In addition, luteolin-7-glucoside promoted hepatic lipid metabolism by inhibiting the activity of HMG CoA reductase in a dose-dependent manner. *G. biloba* flavonoids may help prevent metabolic disease (93, 94). In addition, pretreatment with *G. biloba* extract and quercetin inhibited NO release in a dose-dependent manner (95).

Metabolomics is an omics technique for quantitatively analyzing all endogenous metabolites in organisms and finding the relative relationship between the metabolites and physiological and pathological changes. This is in line with the integrity and systemic characteristics of traditional Chinese medicine. This technology can provide good support for uncovering the cardioprotective mechanism of *G. biloba* and facilitating the quality control of *G. biloba* extract. The general method of identifying endogenous metabolites relies on the use of liquid chromatography mass spectrometry and gas chromatography mass spectrometry to analyze biological samples such as plasma and urine. One work (96) employed ultra-high performance liquid chromatography tandem with quadrupole time-of-flight mass spectrometry to identify eighteen serum endogenous metabolites related to *G. biloba* extract’s protective effect against positive acceleration exposure, mainly involving fatty acid oxidation, glycerophospholipid metabolism, and phospholipid metabolism, bile acid metabolism, purine metabolism and lysine metabolism, and other pathways.

However, the use of small samples to develop a suitable analysis platform that can simultaneously cover enough endogenous metabolites related to multiple metabolic pathways is still the bottleneck of metabolomics research. First, the polarity of the endogenous metabolites is significantly different, so the sample preparation is also different; Second, the abundance of endogenous metabolites is very different, the mass detection method is more conducive to the detection of high abundance ions. This leads to the omission of low-abundance metabolites in plasma, urine, and target tissues. Third, the number of samples in animal and clinical experiments is always small and precious, and it is necessary to use small samples to obtain as much information as possible.

To overcome these hurdles, one group (97) improved the efficiency and capacity of the electrospray ionization method by spiking ammonium formate into the mobile phase, and only 20 μL aliquots of plasma samples were required to simultaneously determine the ginkgo flavonoids and terpenoids in plasma within 5 min. This method greatly improves the sensitivity, reduces the matrix effect, expands the linear range, and shortens the detection time. In order to simultaneously characterize lipids and polar metabolites of different intensities, another group (98) developed a new liquid-liquid extraction method to extract and separate target analytes from micro-samples (100 μL), and then these endogenous metabolites, i.e., sphingolipids, phosphoglycerides, glycerides, and sphingomyelins were analyzed on a triple quadrupole mass spectrometer. At the same time, a targeted metabolomics analysis platform was developed. Notably, only 100 μL of biological samples were used to quantify 808 endogenous metabolites, covering the core network of lipid, glucose, amino acid, and nucleotide metabolism. This platform was employed for metabolic profiling of endogenous metabolites of myocardial ischemia-reperfusion rats and *G. biloba* extract-preconditioned rats. Forty-seven metabolites were discovered as potential biomarkers. After myocardial ischemia-reperfusion, oxidative stress, and structural damage lead to metabolic disorders. *G. biloba* extract can effectively restore the levels of fatty acids, sphingolipids, phosphoglycerides, glycerides, amino acids, and energy metabolism, which is closely related to its antioxidant, platelet-activating factor antagonistic and lipid-lowering properties.

Due to the poor absorption of natural medicines and the low concentration of metabolites, its metabolomic characterization is still extremely challenging. Complex biological matrices often affect the determination results of low-concentration metabolites. Cao et al. (99) developed a strategy called intestinal mucosal metabolome guided detection (IMMD) to solve this problem. The basic principle is that poorly absorbed natural products are usually concentrated and extensively metabolized by intestinal cells before entering the bloodstream and being distributed to other organs. First, the metabolites in rat intestinal mucosa after treatment with *G. biloba* extract were identified, and then the identified metabolome was used as a target database for metabolomics analysis of rat plasma, liver and brain. Finally, the IMMD strategy was applied to identify 39, 45, and 6 metabolites in plasma, liver, and brain, respectively. Therefore, the IMMD strategy provides higher sensitivity and specificity for detecting low-abundance compounds in complex mixtures.

Commercial preparations of *G. biloba* are complex mixtures prepared from raw leaf extracts through a series of extraction and pre-purification steps. The quality of *G. biloba* preparations is uneven, and the quality of *G. biloba* preparations will seriously affect the efficacy of the drug (100). To standardize *G. biloba* preparations, one group (101) developed a ^1^H NMR-based metabolomics method and combined high-performance liquid chromatography, photodiode array detection, mass spectrometry, solid-phase extraction, and nuclear magnetic resonance for analyze 16 commercially available *G. biloba* preparations, which were collected from Denmark, Italy, Sweden, and the United Kingdom. Eight secondary metabolites were identified as quality markers.

## Conclusion

Existing clinical practice and experimental studies have confirmed that *G. biloba* flavonoids and their preparations have cardioprotective effects, and are clinically used to treat cardiovascular diseases such as coronary heart disease, hypertension, angina pectoris, and atherosclerosis. Quercetin, kaempferol, apigenin, and luteolin of *G. biloba* flavonoids are heavily studied, which have been proven to protect myocardial ischemia-reperfusion injury, protect endothelial cells, and prevent coronary atherosclerosis, but other bioactive flavonoids of are less studied. Single flavonoid of *G. biloba* is less effective than *G. biloba* extracts. Therefore, it is important to explore other bioactive flavonoids of *G. biloba* and the interaction between different flavonoids of *G. biloba*.

Due to the first-pass effect and glucuronidation, the oral bioavailability of flavonoids of *G. biloba* is low. The metabolism of flavonoids is mainly mediated by UGT1A9 enzyme in the liver. The glucuronidation and hydrolysis of flavonoids into aglycones are accomplished under the fermentation of the intestinal flora in the small intestine. The major form of glucuronic acid or sulfate adduct of flavonoids were observed in plasma and urine. Novel drug delivery systems such as liposomes, phospholipid complexes and solid dispersions of *G. biloba* flavonoids can improve the bioavailability.

The gut microbiota is a complex group of microorganisms that exist in the gastrointestinal tract in a symbiotic manner with the host. The intra-individual composition of the gut microbiota is dynamic, and the inter-individual composition of the gut microbiota also varies dramatically. Therefore, the therapeutical effect of the same drug at the same dosage will be different between individuals. Precision medicine, which takes differences in individual genes, environment and living habits into account, has become the mainstay of modern therapy and provides an emerging strategy for prevention and treatment of diseases. Individualized medication can achieve the most important path to precision medicine. Undoubtedly, precise dose adjustment based on the composition of the fecal gut microbiota is preferred (102, 103). The timing of administration should also be considered, as circadian rhythms play a crucial role in fluctuations in the composition and function of gut microbiota, which can significantly affect drug efficacy by modulating gut microbiota metabolites. Targeting the gut microbiota through a combination of drugs, which target bacterial enzymes such as beta-glucuronidase that is responsible for drug transformation, is also a good option of future precision medicine (104).

The metabolomic investigation of *G. biloba* flavonoids shows that there are still several problems and challenges in the application of this method. For example, the physical and chemical properties of different metabolites are very different and cannot be extracted at the same time. Most metabolites are very low in the body fluids, making it difficult to quantify. The current metabolomics database is not perfect, and there are still many endogenous metabolites whose structures cannot be confirmed. If these problems can be overcome in the future, metabolomics will play a huge role in comprehensive understanding of the cardioprotective effect of *G. biloba* flavonoids.

Collectively, this review summarized the bioactive flavonoids of *G. biloba* and presented metabolism, pharmacokinetics and metabolomic studies for elucidating its mechanism of action on cardiovascular diseases, and these studies will be helpful for understanding the cardioprotective effect of *G. biloba* flavonoids.

## Author Contributions

All authors listed have made a substantial, direct, and intellectual contribution to the work, and approved it for publication.

## Conflict of Interest

The authors declare that the research was conducted in the absence of any commercial or financial relationships that could be construed as a potential conflict of interest.

## Publisher’s Note

All claims expressed in this article are solely those of the authors and do not necessarily represent those of their affiliated organizations, or those of the publisher, the editors and the reviewers. Any product that may be evaluated in this article, or claim that may be made by its manufacturer, is not guaranteed or endorsed by the publisher.

## References

[1] Soehnlein O Libby P. Targeting inflammation in atherosclerosis — from experimental insights to the clinic. *Nat Rev Drug Discov.* (2021) 20:589–610. 10.1038/s41573-021-00198-1 33976384 PMC8112476

[2] Lundberg JO Gladwin MT Weitzberg E. Strategies to increase nitric oxide signalling in cardiovascular disease. *Nat Rev Drug Discov.* (2015) 14:623–41. 10.1038/nrd4623 26265312

[3] Liu H Wang X Wang G Cui P Wu S Ai C The nearly complete genome of *Ginkgo biloba* illuminates gymnosperm evolution. *Nat Plants.* (2021) 7:748–56. 10.1038/s41477-021-00933-x 34135482

[4] Krammer F. On the therapy of peripheral circulatory disorders with the new angioactivator tebonin of plant origin. *Med Welt.* (1966) 28:1524–8. 5984513

[5] Peterson JJ Dwyer JT Jacques PF McCullough ML. Associations between flavonoids and cardiovascular disease incidence or mortality in European and US populations. *Nutr Rev.* (2012) 70:491–508. 10.1111/j.1753-4887.2012.00508.x 22946850 PMC4130174

[6] Satoh H Nishida S. Electropharmacological actions of *Ginkgo biloba* extract on vascular smooth and heart muscles. *Clin Chim Acta.* (2004) 342:13–22. 10.1016/j.cccn.2003.12.014 15026263

[7] Yang Y Li Y Wang J Sun K Tao W Wang Z Systematic investigation of *Ginkgo biloba* leaves for treating cardio-cerebrovascular diseases in an animal model. *ACS Chem Biol.* (2017) 12:1363–72. 10.1021/acschembio.6b00762 28333443

[8] Wei T Xiong FF Wang SD Wang K Zhang YY Zhang QH. Flavonoid ingredients of *Ginkgo biloba* leaf extract regulate lipid metabolism through Sp1-mediated carnitine palmitoyltranferase 1A up-regulation. *J Biomed Sci.* (2014) 21:87. 10.1186/s12929-014-0087-x 25183267 PMC4428510

[9] Du Y Xu BJ Deng X Wu XW Li YJ Wang SR Predictive metabolic signatures for the occurrence and development of diabetic nephropathy and the intervention of *Ginkgo biloba* leaves extract based on gas or liquid chromatography with mass spectrometry. *J Pharm Biomed Anal.* (2019) 166:30–9. 10.1016/j.jpba.2018.12.017 30599279

[10] Zheng X Gao Q Liang S Zhu G Wang D Feng Y. Cardioprotective properties of *Ginkgo biloba* extract 80 via the activation of AKT/GSK3β/β-Catenin signaling pathway. *Front Mol Biosci.* (2021) 8:771208. 10.3389/fmolb.2021.771208 34805278 PMC8595256

[11] Trumbeckaite S Bernatoniene J Majiene D Jakstas V Savickas A Toleikis A. Effect of *Ginkgo biloba* extract on the rat heart mitochondrial function. *J Ethnopharmacol.* (2007) 111:512–6. 10.1016/j.jep.2006.12.028 17258877

[12] Li T Zhang Y Tian J Yang L Wang J. *Ginkgo biloba* pretreatment attenuates myocardial ischemia-reperfusion injury via mitoBK(Ca). *Am J Chin Med.* (2019) 47:1057–73. 10.1142/s0192415x1950054x 31327236

[13] Song W Zhao J Yan XS Fang X Huo DS Wang H Mechanisms associated with protective effects of *Ginkgo Biloba* leaf extracton in rat cerebral ischemia reperfusion injury. *J Toxicol Environ Health A.* (2019) 82:1045–51. 10.1080/15287394.2019.1686215 31735125

[14] Huang XF Zhang SZ You YY Zhang N Lu H Daugherty A *Ginkgo biloba* extracts prevent aortic rupture in angiotensin II-infused hypercholesterolemic mice. *Acta Pharmacol Sin.* (2019) 40:192–8. 10.1038/s41401-018-0017-7 29777203 PMC6329785

[15] Liu L Wang Y Zhang J Wang S. Advances in the chemical constituents and chemical analysis of *Ginkgo biloba* leaf, extract, and phytopharmaceuticals. *J Pharm Biomed Anal.* (2021) 193:113704. 10.1016/j.jpba.2020.113704 33157480

[16] Ude C Schubert-Zsilavecz M Wurglics M. *Ginkgo biloba* extracts: a review of the pharmacokinetics of the active ingredients. *Clin Pharmacokinet.* (2013) 52:727–49. 10.1007/s40262-013-0074-5 23703577

[17] Yamagata K Hashiguchi K Yamamoto H Tagami M. Dietary Apigenin reduces induction of LOX-1 and NLRP3 expression, leukocyte adhesion, and acetylated low-density Lipoprotein uptake in human endothelial cells exposed to Trimethylamine-N-Oxide. *J Cardiovasc Pharmacol.* (2019) 74:558–65. 10.1097/fjc.0000000000000747 31815868

[18] Clayton ZS Hutton DA Brunt VE VanDongen NS Ziemba BP Casso AG Apigenin restores endothelial function by ameliorating oxidative stress, reverses aortic stiffening, and mitigates vascular inflammation with aging. *Am J Physiol Heart Circ Physiol.* (2021) 321:H185–96. 10.1152/ajpheart.00118.2021 34114892 PMC8321807

[19] Wang F Fan K Zhao Y Xie ML. Apigenin attenuates TGF-β1-stimulated cardiac fibroblast differentiation and extracellular matrix production by targeting miR-155-5p/c-Ski/Smad pathway. *J Ethnopharmacol.* (2021) 265:113195. 10.1016/j.jep.2020.113195 32800930

[20] Li W Chen L Xiao Y. Apigenin protects against ischemia-/hypoxia-induced myocardial injury by mediating pyroptosis and apoptosis. *In Vitro Cell Dev Biol Anim.* (2020) 56:307–12. 10.1007/s11626-020-00434-9 32406012

[21] Kowalska I Adach W Stochmal A Olas B. A comparison of the effects of apigenin and seven of its derivatives on selected biomarkers of oxidative stress and coagulation in vitro. *Food Chem Toxicol.* (2020) 136:111016. 10.1016/j.fct.2019.111016 31805303

[22] Li D Ma J Wang L Xin S. Apigenin prevent abdominal aortic aneurysms formation by inhibiting the NF-κB signaling pathway. *J Cardiovasc Pharmacol.* (2020) 75:229–39. 10.1097/fjc.0000000000000785 31821190

[23] Zhou Z Zhang Y Lin L Zhou J. Apigenin suppresses the apoptosis of H9C2 rat cardiomyocytes subjected to myocardial ischemia-reperfusion injury via upregulation of the PI3K/Akt pathway. *Mol Med Rep.* (2018) 18:1560–70. 10.3892/mmr.2018.9115 29901074 PMC6072196

[24] Janssen K Mensink RP Cox FJ Harryvan JL Hovenier R Hollman PC Effects of the flavonoids quercetin and apigenin on hemostasis in healthy volunteers: results from an in vitro and a dietary supplement study. *Am J Clin Nutr.* (1998) 67:255–62. 10.1093/ajcn/67.2.255 9459373

[25] Basu A Das AS Majumder M Mukhopadhyay R. Antiatherogenic Roles of Dietary Flavonoids Chrysin, Quercetin, and Luteolin. *J Cardiovasc Pharmacol.* (2016) 68:89–96. 10.1097/fjc.0000000000000380 27385185

[26] Ding X Zheng L Yang B Wang X Ying Y. Luteolin attenuates atherosclerosis via modulating signal transducer and activator Of transcription 3-mediated inflammatory response. *Drug Des Devel Ther.* (2019) 13:3899–911. 10.2147/dddt.s207185 31819365 PMC6874161

[27] Qian LB Wang HP Chen Y Chen FX Ma YY Bruce IC Luteolin reduces high glucose-mediated impairment of endothelium-dependent relaxation in rat aorta by reducing oxidative stress. *Pharmacol Res.* (2010) 61:281–7. 10.1016/j.phrs.2009.10.004 19892019

[28] Assunção HCR Cruz YMC Bertolino JS Garcia RCT Fernandes L. Protective effects of luteolin on the venous endothelium. *Mol Cell Biochem.* (2021) 476:1849–59. 10.1007/s11010-020-04025-w 33469821

[29] Oyagbemi AA Omobowale TO Ola-Davies OE Asenuga ER Ajibade TO Adejumobi OA Luteolin-mediated Kim-1/NF-kB/Nrf2 signaling pathways protects sodium fluoride-induced hypertension and cardiovascular complications. *Biofactors.* (2018) 44:518–31. 10.1002/biof.1449 30474894

[30] Li W Dong M Guo P Liu Y Jing Y Chen R Luteolin-induced coronary arterial relaxation involves activation of the myocyte voltage-gated K(+) channels and inward rectifier K(+) channels. *Life Sci.* (2019) 221:233–40. 10.1016/j.lfs.2019.02.028 30771310

[31] Chen HI Hu WS Hung MY Ou HC Huang SH Hsu PT Protective effects of luteolin against oxidative stress and mitochondrial dysfunction in endothelial cells. *Nutr Metab Cardiovasc Dis.* (2020) 30:1032–43. 10.1016/j.numecd.2020.02.014 32402583

[32] Xu T Li D Jiang D. Targeting cell signaling and apoptotic pathways by luteolin: cardioprotective role in rat cardiomyocytes following ischemia/reperfusion. *Nutrients.* (2012) 4:2008–19. 10.3390/nu4122008 23235403 PMC3546619

[33] Yang JT Qian LB Zhang FJ Wang J Ai H Tang LH Cardioprotective effects of luteolin on ischemia/reperfusion injury in diabetic rats are modulated by eNOS and the mitochondrial permeability transition pathway. *J Cardiovasc Pharmacol.* (2015) 65:349–56. 10.1097/fjc.0000000000000202 25502309

[34] Wang Y Xie X Liu L Zhang H Ni F Wen J Four new flavonol glycosides from the leaves of *Ginkgo biloba*. *Nat Prod Res.* (2021) 35:2520–5. 10.1080/14786419.2019.1684282 31680566

[35] Ban C Park JB Cho S Kim HR Kim YJ Bae H Characterization of *Ginkgo biloba* leaf flavonoids as neuroexocytosis regulators. *Molecules.* (2020) 25:1829. 10.3390/molecules25081829 32316426 PMC7221681

[36] Lan WJ Zheng XX. Activity of Ginkgo biloba extract and quercetin on thrombomodulin expression and tissue-type plasminogen activator secretion by human umbilical vein endothelial cells. *Biomed Environ Sci.* (2006) 19:249–53. 17044640

[37] Nishida S Satoh H. Comparative vasodilating actions among terpenoids and flavonoids contained in *Ginkgo biloba* extract. *Clin Chim Acta.* (2004) 339:129–33. 10.1016/j.cccn.2003.10.004 14687903

[38] Hu WH Wang HY Xia YT Dai DK Xiong QP Dong TT Kaempferol, a major flavonoid in *Ginkgo* folium, potentiates angiogenic functions in cultured endothelial cells by binding to vascular endothelial growth factor. *Front Pharmacol.* (2020) 11:526. 10.3389/fphar.2020.00526 32410995 PMC7198864

[39] Gan L Ma J You G Mai J Wang Z Yang R Glucuronidation and its effect on the bioactivity of amentoflavone, a biflavonoid from *Ginkgo biloba* leaves. *J Pharm Pharmacol.* (2020) 72:1840–53. 10.1111/jphp.13247 32144952

[40] Chen TR Wei LH Guan XQ Huang C Liu ZY Wang FJ Biflavones from *Ginkgo biloba* as inhibitors of human thrombin. *Bioorg Chem.* (2019) 92:103199. 10.1016/j.bioorg.2019.103199 31446241

[41] Mika D Bobin P Pomérance M Lechêne P Westenbroek RE Catterall WA Differential regulation of cardiac excitation-contraction coupling by cAMP phosphodiesterase subtypes. *Cardiovasc Res.* (2013) 100:336–46. 10.1093/cvr/cvt193 23933582 PMC3888219

[42] Saponara R Bosisio E. Inhibition of cAMP-phosphodiesterase by biflavones of *Ginkgo biloba* in rat adipose tissue. *J Nat Prod.* (1998) 61:1386–7. 10.1021/np970569m 9834158

[43] Dell’Agli M Galli GV Bosisio E. Inhibition of cGMP-phosphodiesterase-5 by biflavones of *Ginkgo biloba*. *Planta Med.* (2006) 72:468–70. 10.1055/s-2005-916236 16557462

[44] Wu J Xu X Li Y Kou J Huang F Liu B Quercetin, luteolin and epigallocatechin gallate alleviate TXNIP and NLRP3-mediated inflammation and apoptosis with regulation of AMPK in endothelial cells. *Eur J Pharmacol.* (2014) 745:59–68. 10.1016/j.ejphar.2014.09.046 25446924

[45] Li CL Wong YY. The bioavailability of ginkgolides in *Ginkgo biloba* extracts. *Planta Med.* (1997) 63:563–5. 10.1055/s-2006-957768 9434615

[46] Erlund I Kosonen T Alfthan G Mäenpää J Perttunen K Kenraali J Pharmacokinetics of quercetin from quercetin aglycone and rutin in healthy volunteers. *Eur J Clin Pharmacol.* (2000) 56:545–53. 10.1007/s002280000197 11151743

[47] Pietta PG Gardana C Mauri PL. Identification of *Gingko biloba* flavonol metabolites after oral administration to humans. *J Chromatogr B Biomed Sci Appl.* (1997) 693:249–55. 10.1016/s0378-4347(96)00513-0 9200545

[48] Wang FM Yao TW Zeng S. Determination of quercetin and kaempferol in human urine after orally administrated tablet of *Ginkgo biloba* extract by HPLC. *J Pharm Biomed Anal.* (2003) 33:317–21. 10.1016/s0731-7085(03)00255-3 12972097

[49] Watson DG Oliveira EJ. Solid-phase extraction and gas chromatography-mass spectrometry determination of kaempferol and quercetin in human urine after consumption of *Ginkgo biloba* tablets. *J Chromatogr B Biomed Sci Appl.* (1999) 723:203–10. 10.1016/s0378-4347(98)00509-x 10080647

[50] Moreau JP Eck CR McCabe J Skinner S. Absorption, distribution and elimination of a labelled extract of *Ginkgo biloba* leaves in the rat. *Presse Med.* (1986) 15:1458–61. 2947082

[51] Rao Z Qin H Wei Y Zhou Y Zhang G Zhang F Development of a dynamic multiple reaction monitoring method for determination of digoxin and six active components of *Ginkgo biloba* leaf extract in rat plasma. *J Chromatogr B Analyt Technol Biomed Life Sci.* (2014) 959:27–35. 10.1016/j.jchromb.2014.03.028 24747521

[52] Wang FM Yao TW Zeng S. Disposition of quercetin and kaempferol in human following an oral administration of *Ginkgo biloba* extract tablets. *Eur J Drug Metab Pharmacokinet.* (2003) 28:173–7. 10.1007/bf03190482 14527089

[53] Wang Y Cao J Zeng S. Involvement of P-glycoprotein in regulating cellular levels of *Ginkgo* flavonols: quercetin, kaempferol, and isorhamnetin. *J Pharm Pharmacol.* (2005) 57:751–8. 10.1211/0022357056299 15969930

[54] An G Gallegos J Morris ME. The bioflavonoid kaempferol is an Abcg2 substrate and inhibits Abcg2-mediated quercetin efflux. *Drug Metab Dispos.* (2011) 39:426–32. 10.1124/dmd.110.035212 21139040

[55] Wang T Xiao J Hou H Li P Yuan Z Xu H Development of an ultra-fast liquid chromatography-tandem mass spectrometry method for simultaneous determination of seven flavonoids in rat plasma: application to a comparative pharmacokinetic investigation of *Ginkgo biloba* extract and single pure *Ginkgo* flavonoids after oral administration. *J Chromatogr B Analyt Technol Biomed Life Sci.* (2017) 1060:173–81. 10.1016/j.jchromb.2017.05.021 28622621

[56] Murota K Nakamura Y Uehara M. Flavonoid metabolism: the interaction of metabolites and gut microbiota. *Biosci Biotechnol Biochem.* (2018) 82:600–10. 10.1080/09168451.2018.1444467 29504827

[57] Chen Y Xie S Chen S Zeng S. Glucuronidation of flavonoids by recombinant UGT1A3 and UGT1A9. *Biochem Pharmacol.* (2008) 76:416–25. 10.1016/j.bcp.2008.05.007 18565494

[58] Xie S Chen Y Chen S Zeng S. Structure-metabolism relationships for the glucuronidation of flavonoids by UGT1A3 and UGT1A9. *J Pharm Pharmacol.* (2011) 63:297–304. 10.1111/j.2042-7158.2010.01168.x 21235595

[59] Mohamed MF Frye RF. Inhibition of intestinal and hepatic glucuronidation of mycophenolic acid by *Ginkgo biloba* extract and flavonoids. *Drug Metab Dispos.* (2010) 38:270–5. 10.1124/dmd.109.030080 19889883

[60] Mohamed ME Frye RF. Inhibitory effects of commonly used herbal extracts on UDP-glucuronosyltransferase 1A4, 1A6, and 1A9 enzyme activities. *Drug Metab Dispos.* (2011) 39:1522–8. 10.1124/dmd.111.039602 21632963 PMC3164271

[61] Chang TK Chen J Teng XW. Distinct role of bilobalide and ginkgolide A in the modulation of rat CYP2B1 and CYP3A23 gene expression by *Ginkgo biloba* extract in cultured hepatocytes. *Drug Metab Dispos.* (2006) 34:234–42. 10.1124/dmd.105.005751 16258077

[62] Deng Y Bi HC Zhao LZ He F Liu YQ Yu JJ Induction of cytochrome P450s by terpene trilactones and flavonoids of the *Ginkgo biloba* extract EGb 761 in rats. *Xenobiotica.* (2008) 38:465–81. 10.1080/00498250701883233 18421621

[63] Lau AJ Chang TK. Inhibition of human CYP2B6-catalyzed bupropion hydroxylation by *Ginkgo biloba* extract: effect of terpene trilactones and flavonols. *Drug Metab Dispos.* (2009) 37:1931–7. 10.1124/dmd.109.028118 19487249

[64] Ribonnet L Callebaut A Nobels I Scippo ML Schneider YJ De Saeger S Modulation of CYP1A1 activity by a *Ginkgo biloba* extract in the human intestinal Caco-2 cells. *Toxicol Lett.* (2011) 202:193–202. 10.1016/j.toxlet.2011.02.006 21329749

[65] Feltrin C Farias IV Sandjo LP Reginatto FH Simões CMO. Effects of standardized medicinal plant extracts on drug metabolism mediated by CYP3A4 and CYP2D6 enzymes. *Chem Res Toxicol.* (2020) 33:2408–19. 10.1021/acs.chemrestox.0c00182 32786546

[66] Loretz C Ho MD Alam N Mitchell W Li AP. Application of cryopreserved human intestinal mucosa and cryopreserved human enterocytes in the evaluation of herb-drug interactions: evaluation of CYP3A inhibitory potential of grapefruit juice and commercial formulations of twenty-nine herbal supplements. *Drug Metab Dispos.* (2020) 48:1084–91. 10.1124/dmd.120.000033 32719085

[67] Chen Y Xiao P Ou-Yang DS Fan L Guo D Wang YN Simultaneous action of the flavonoid quercetin on cytochrome P450 (CYP) 1A2, CYP2A6, N-acetyltransferase and xanthine oxidase activity in healthy volunteers. *Clin Exp Pharmacol Physiol.* (2009) 36:828–33. 10.1111/j.1440-1681.2009.05158.x 19215233

[68] Palle S Neerati P. Quercetin nanoparticles alter pharmacokinetics of bromocriptine, reflecting its enhanced inhibitory action on liver and intestinal CYP 3A enzymes in rats. *Xenobiotica.* (2018) 48:1028–36. 10.1080/00498254.2017.1390277 28990837

[69] Quintieri L Palatini P Nassi A Ruzza P Floreani M. Flavonoids diosmetin and luteolin inhibit midazolam metabolism by human liver microsomes and recombinant CYP 3A4 and CYP3A5 enzymes. *Biochem Pharmacol.* (2008) 75:1426–37. 10.1016/j.bcp.2007.11.012 18191104

[70] Kitakaze T Makiyama A Samukawa Y Jiang S Yamashita Y Ashida H. A physiological concentration of luteolin induces phase II drug-metabolizing enzymes through the ERK1/2 signaling pathway in HepG2 cells. *Arch Biochem Biophys.* (2019) 663:151–9. 10.1016/j.abb.2019.01.012 30641047

[71] Lee HS Kim MJ. Selective responses of three *Ginkgo biloba* leaf-derived constituents on human intestinal bacteria. *J Agric Food Chem.* (2002) 50:1840–4. 10.1021/jf011140a 11902921

[72] Liu DY Zhu HJ Zheng YF Zhu XQ. Kaempferol activates human steroid and xenobiotic receptor-mediated cytochrome P450 3A4 transcription. *J Zhejiang Univ.* (2006) 35:14–7. 10.3785/j.issn.1008-9292.2006.01.003 16470914

[73] Liu J Tan Y Cheng H Zhang D Feng W Peng C. Functions of gut microbiota metabolites, current status and future perspectives. *Aging Dis.* (2022). 10.14336/ad.2022.0104 PMC9286904 35855347

[74] Feng W Ao H Peng C Yan D. Gut microbiota, a new frontier to understand traditional Chinese medicines. *Pharmacol Res.* (2019) 142:176–91. 10.1016/j.phrs.2019.02.024 30818043

[75] Weng ZM Wang P Ge GB Dai ZR Wu DC Zou LW Structure-activity relationships of flavonoids as natural inhibitors against E. coli β-glucuronidase. *Food Chem Toxicol.* (2017) 109:975–83. 10.1016/j.fct.2017.03.042 28347758

[76] Bai Y Chen L Wang PP Tang YQ Wu DC Zhang CL Discovery of a naturally occurring broad-spectrum inhibitor against gut bacterial β-glucuronidases from *Ginkgo biloba*. *Food Funct.* (2021) 12:11190–201. 10.1039/d1fo01748a 34668903

[77] Lin W Wang W Yang H Wang D Ling W. Influence of intestinal microbiota on the catabolism of flavonoids in mice. *J Food Sci.* (2016) 81:H3026–34. 10.1111/1750-3841.13544 27792839

[78] Zhang F He F Li L Guo L Zhang B Yu S Bioavailability based on the gut microbiota: a new perspective. *Microbiol Mol Biol Rev.* (2020) 84:e00072-19. 10.1128/MMBR.00072-19 32350027 PMC7194497

[79] Hanske L Loh G Sczesny S Blaut M Braune A. The bioavailability of apigenin-7-glucoside is influenced by human intestinal microbiota in rats. *J Nutr.* (2009) 139:1095–102. 10.3945/jn.108.102814 19403720

[80] Tan X Sun Z Ye C. Dietary Ginkgo biloba leaf extracts supplementation improved immunity and intestinal morphology, antioxidant ability and tight junction proteins mRNA expression of hybrid groupers (*Epinephelus lanceolatus* ♁ × *Epinephelus fuscoguttatus* ♀) fed high lipid diets. *Fish Shellfish Immunol.* (2020) 98:611–8. 10.1016/j.fsi.2019.09.034 31533081

[81] Qiao Y Zhang Z Zhai Y Yan X Zhou W Liu H Apigenin Alleviates Obesity-associated metabolic syndrome by regulating the composition of the gut microbiome. *Front Microbiol.* (2021) 12:805827. 10.3389/fmicb.2021.805827 35046924 PMC8762173

[82] Oh S Koike S Kobayashi Y. Effect of *Ginkgo* extract supplementation on in vitro rumen fermentation and bacterial profiles under different dietary conditions. *Anim Sci J.* (2017) 88:1737–43. 10.1111/asj.12877 28707415

[83] Kim J-K Choi MS Kim J-Y Yu JS Seo JI Yoo HH *Ginkgo biloba* leaf extract suppresses intestinal human breast cancer resistance protein expression in mice: correlation with gut microbiota. *Biomed Pharmacother.* (2021) 140:111712. 10.1016/j.biopha.2021.111712 34010745

[84] Morand C Manach C Crespy V Remesy C. Respective bioavailability of quercetin aglycone and its glycosides in a rat model. *Biofactors.* (2000) 12:169–74. 10.1002/biof.5520120127 11216481

[85] Bedir E Tatli II Khan RA Zhao J Takamatsu S Walker LA Biologically active secondary metabolites from *Ginkgo biloba*. *J Agric Food Chem.* (2002) 50:3150–5. 10.1021/jf011682s 12009978

[86] Hollman PC Bijsman MN van Gameren Y Cnossen EP de Vries JH Katan MB. The sugar moiety is a major determinant of the absorption of dietary flavonoid glycosides in man. *Free Radic Res.* (1999) 31:569–73. 10.1080/10715769900301141 10630681

[87] Liao S Ren Q Yang C Zhang T Li J Wang X Liquid chromatography-tandem mass spectrometry determination and pharmacokinetic analysis of amentoflavone and its conjugated metabolites in rats. *J Agric Food Chem.* (2015) 63:1957–66. 10.1021/jf5019615 25415840

[88] Zheng XX Du Y Xu BJ Wang TY Zhong QQ Li Z Off-line two-dimensional liquid chromatography coupled with diode array detection and quadrupole-time of flight mass spectrometry for the biotransformation kinetics of *Ginkgo biloba* leaves extract by diabetic rat liver microsomes. *J Chromatogr B Analyt Technol Biomed Life Sci.* (2019) 1109:1–9. 10.1016/j.jchromb.2019.01.015 30690396

[89] Zheng B Xing G Bi Y Yan G Wang J Cheng Y Comparative pharmacokinetics of a proliposome formulation of *Ginkgo biloba* extract and Ginaton in rats by a sensitive ultra performance liquid chromatography-tandem mass spectrometry method. *Saudi J Biol Sci.* (2016) 23:54–65. 10.1016/j.sjbs.2015.08.009 26858539 PMC4705248

[90] Chen ZP Sun J Chen HX Xiao YY Liu D Chen J Comparative pharmacokinetics and bioavailability studies of quercetin, kaempferol and isorhamnetin after oral administration of *Ginkgo biloba* extracts, *Ginkgo biloba* extract phospholipid complexes and *Ginkgo biloba* extract solid dispersions in rats. *Fitoterapia.* (2010) 81:1045–52. 10.1016/j.fitote.2010.06.028 20603197

[91] Hoek-van den Hil EF van Schothorst EM van der Stelt I Swarts HJ van Vliet M Amolo T Direct comparison of metabolic health effects of the flavonoids quercetin, hesperetin, epicatechin, apigenin and anthocyanins in high-fat-diet-fed mice. *Genes Nutr.* (2015) 10:469. 10.1007/s12263-015-0469-z 26022682 PMC4447677

[92] Duarte S Arango D Parihar A Hamel P Yasmeen R Doseff AI. Apigenin protects endothelial cells from lipopolysaccharide (LPS)-induced inflammation by decreasing caspase-3 activation and modulating mitochondrial function. *Int J Mol Sci.* (2013) 14:17664–79. 10.3390/ijms140917664 23989609 PMC3794747

[93] Sá C Oliveira AR Machado C Azevedo M Pereira-Wilson C. Effects on liver lipid metabolism of the naturally occurring dietary flavone luteolin-7-glucoside. *Evid Based Complement Alternat Med.* (2015) 2015:647832. 10.1155/2015/647832 26113868 PMC4465769

[94] Azevedo MF Camsari C Sá CM Lima CF Fernandes-Ferreira M Pereira-Wilson C. Ursolic acid and luteolin-7-glucoside improve lipid profiles and increase liver glycogen content through glycogen synthase kinase-3. *Phytother Res.* (2010) 24(Suppl. 2):S220–4. 10.1002/ptr.3118 20127879

[95] Wadsworth TL Koop DR. Effects of *Ginkgo biloba* extract (EGb 761) and quercetin on lipopolysaccharide-induced release of nitric oxide. *Chem Biol Interact.* (2001) 137:43–58. 10.1016/s0009-2797(01)00208-3 11518563

[96] Yang Z Zhao A Li Z Ge H Li T Zhang F Metabolomics reveals positive acceleration(+Gz)-induced metabolic perturbations and the protective effect of *Ginkgo biloba* extract in a rat model based on ultra high-performance liquid chromatography coupled with quadrupole time-of-flight mass spectrometry. *J Pharm Biomed Anal.* (2016) 125:77–84. 10.1016/j.jpba.2016.03.016 27010354

[97] Zhao Y Sun Y Li C. Simultaneous determination of ginkgo flavonoids and terpenoids in plasma: ammonium formate in LC mobile phase enhancing electrospray ionization efficiency and capacity. *J Am Soc Mass Spectrom.* (2008) 19:445–9. 10.1016/j.jasms.2007.11.015 18155919

[98] Wang Z Zhang J Ren T Dong Z. Targeted metabolomic profiling of cardioprotective effect of *Ginkgo biloba* L. extract on myocardial ischemia in rats. *Phytomedicine.* (2016) 23:621–31. 10.1016/j.phymed.2016.03.005 27161403

[99] Cao G Wang N He D Wang X Tian Y Wan N Intestinal mucosal metabolites-guided detection of trace-level *Ginkgo biloba* extract metabolome. *J Chromatogr A.* (2019) 1608:460417. 10.1016/j.chroma.2019.460417 31416627

[100] Napolitano JG Gödecke T Rodríguez-Brasco MF Jaki BU Chen SN Lankin DC The tandem of full spin analysis and qHNMR for the quality control of botanicals exemplified with *Ginkgo biloba*. *J Nat Prod.* (2012) 75:238–48. 10.1021/np200949v 22332915 PMC3388902

[101] Agnolet S Jaroszewski JW Verpoorte R Staerk D. H NMR-based metabolomics combined with HPLC-PDA-MS-SPE-NMR for investigation of standardized *Ginkgo biloba* preparations. *Metabolomics.* (2010) 6:292–302. 10.1007/s11306-009-0195-x 20526353 PMC2874492

[102] Davar D Dzutsev AK McCulloch JA Rodrigues RR Chauvin J-M Morrison RM Fecal microbiota transplant overcomes resistance to anti-PD-1 therapy in melanoma patients. *Science.* (2021) 371:595–602. 10.1126/science.abf3363 33542131 PMC8097968

[103] Baruch EN Youngster I Ben-Betzalel G Ortenberg R Lahat A Katz L Fecal microbiota transplant promotes response in immunotherapy-refractory melanoma patients. *Science.* (2021) 371:602–9. 10.1126/science.abb5920 33303685

[104] Feng W Liu J Ao H Yue S Peng C. Targeting gut microbiota for precision medicine: focusing on the efficacy and toxicity of drugs. *Theranostics.* (2020) 10:11278–301. 10.7150/thno.47289 33042283 PMC7532689

