# Supplementary material for: Pharmacokinetic, Metabolism, and Metabolomic Strategies Provide Deep Insight Into the Underlying Mechanism of Ginkgo biloba Flavonoids in the Treatment of Cardiovascular Disease
Source: Front Nutr. 2022 Mar 23;9:857370. doi: 10.3389/fnut.2022.857370 (PMC8984020; doi:10.3389/fnut.2022.857370)
Supplement: Supplementary file 1 [file Table_1.docx]

**TABLE S1.** **The mechanism of Ginkgo flavonoids in the prevention and treatment of cardiovascular diseases**

| **Compound name** | **Disease** | **Mechanism** | **Ref.** |
| --- | --- | --- | --- |
| apigenin | atherosclerosis | reduce the expression of scavenger receptors and adhesion molecules | (17) |
| apigenin | arterial aging | improve oxidative stress | (18) |
| apigenin | myocardial damage | reduce the expression of  miR-155-5p | (19) |
| apigenin | myocardial damage | mediate pyroptosis and apoptosis. | (20) |
| apigenin | aortic aneurysm | inhibit the NF-κB Signaling Pathway | (21) |
| luteolin | atherosclerosis | manage lipid metabolism | (25) |
| luteolin | atherosclerosis | inhibit signal transducer and activator of STAT3 | (26) |
| luteolin | endothelium-dependent relaxation | reduce oxidative stress | (27) |
| luteolin | venous insufficiency | reduce ROS and improve NO availability | (28) |
| luteolin | hypertension | Kim-1/NF-kB/Nrf2 signaling pathways | (29) |
| luteolin | coronary artery spasm | activate the voltage-gated K(+) channels in muscle cells | (30) |
| quercetin | venous endothelial cell thrombosis | increase the t-PA release | (36) |
| kaempferol | angiogenesis | enhance VEGF-induced VEGFR2 | (38) |
| ginkgetin, isoginkgetin | thrombosis | bind to human thrombin at exosite I | (40) |
| epigallocatechin gallate | endothelial cell damage | alleviate TXNIP with regulation of AMPK | (44) |
