# Supplementary figures and images for: Pharmacokinetic, Metabolism, and Metabolomic Strategies Provide Deep Insight Into the Underlying Mechanism of Ginkgo biloba Flavonoids in the Treatment of Cardiovascular Disease
Source: Front Nutr. 2022 Mar 23;9:857370. doi: 10.3389/fnut.2022.857370 (PMC8984020; doi:10.3389/fnut.2022.857370)

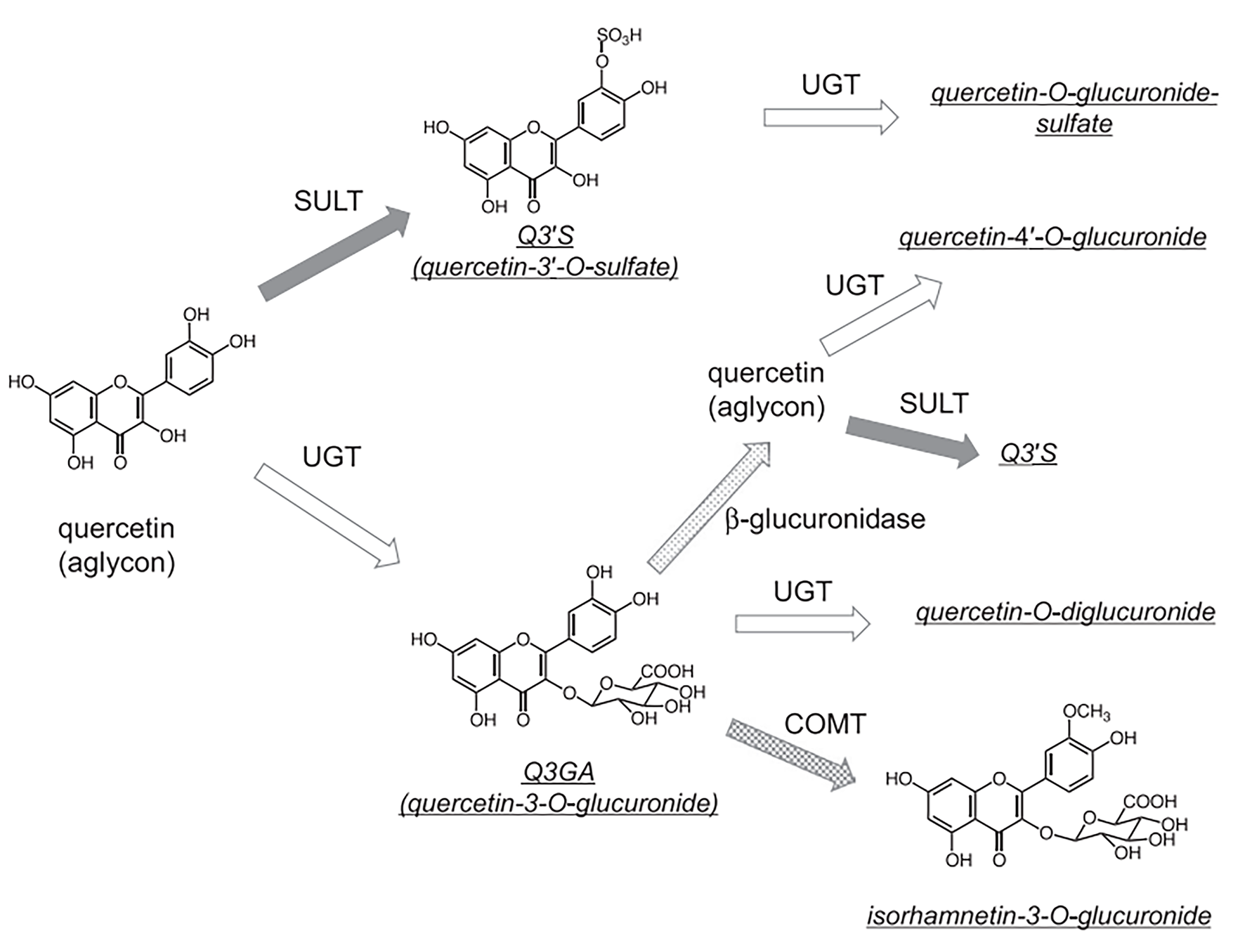

Supplement: Supplementary file 2 [file Image_1.tiff]
